# An outbreak of norovirus-related acute gastroenteritis associated with delivery food in Guangzhou, southern China

**DOI:** 10.1186/s12889-019-8117-y

**Published:** 2020-01-08

**Authors:** Ying Lu, Mengmeng Ma, Hui Wang, Dahu Wang, Chun Chen, Qinlong Jing, Jinmei Geng, Tiegang Li, Zhoubin Zhang, Zhicong Yang

**Affiliations:** 1 Guangzhou Center for Disease Control and Prevention, Guangzhou, Guangdong Province China; 2 Guangzhou Chest Hospital, Guangzhou, Guangdong Province China

**Keywords:** Norovirus, Acute gastroenteritis, Delivery food, Outbreak

## Abstract

**Background:**

A large number of students at a school in Guangzhou city developed a sudden onset of symptoms of diarrhea and vomiting. To help control the outbreak, we conducted an epidemiological investigation to determine the causative agent, sources, role of transmission and risk factors of the infections.

**Methods:**

The study population consisted of probable and confirmed cases. An active search was conducted for cases among all students, teachers and other school staff members. A case control study was carried out using standardized online questionnaires. Data were obtained regarding demographic characteristics, gastrointestinal symptoms, personal hygiene habits, history of contact with a person who had diarrhea and/or vomiting and dining locations during the past 3 days. Rectal swabs or stool samples of the cases and, food handlers, as well as environmental samples were collected to detect potential intestinal viruses and bacteria. We calculated odds ratios and 95% confidence intervals (CIs).

**Results:**

A total of 157 individuals fit the definition of a probable case, including 46 with laboratory-confirmed norovirus infection between March 8 and March 22, 2018. The proportion of students who had eaten delivery food 3 days before the onset of illness in the case group was 2.69 times that in the control group (95%*CI: 1.88–3.85*). Intake of take-out food 3 days earlier, and exposure to similar cases 72 h before onset and case in the same dormitory were risk factors. A total of 20 rectal swab samples from students, 10 rectal swabs from food handlers and 2 environmental swab samples from the out-campus restauranttested positive for norovirus (GII, genogroup II strain).

**Conclusions:**

We investigated an outbreak of norovirus infectious diarrhea. Food handling practices carry potential risk of acute gastroenteritis outbreaks owing to a lack of surveillance and supervision. Greater attention should be paid to the monitoring and supervision of food handlers in off campus restaurant to reduce the incidence of norovirus-related acute gastroenteritis associated with delivery food.

## Background

Noroviruses are a leading cause of sporadic cases and outbreaks of acute gastroenteritis among adults and children, with a substantial impact on public health globally [1, 2].

It can be classified into six genogroups (GI–GVI), three of which (GI, GII, and GIV) cause human disease [3]. Strains of GII.4 genotype have caused 70–80% of all reported outbreaks over the past 13 years [4], and have became the most common cause of outbreaks.

Norovirus are highly contagious and spread rapidly. These viruses can be transmitted through contaminated food or water, directly from person to person, and via aerosol dissemination [5]. Several characteristics make noroviruses challenging to control, which can lead to large outbreaks, such as varied transmission routes, resistance to common disinfectants, low infectious dose, and copious shedding among individuals with asymptomatic infections as well as prior to, during, and after the manifestation of symptomatic infection [6–8].

Food handlers are often suspected as the main source of foodborne outbreaks [9]. Norovirus infection is often associated with contaminated food, and previous studies have suggested that more than 50% of foodborne disease outbreaks are caused by food contaminated with Norovirus [10].

Reported outbreaks predominantly take place in schools, child-care centers, health-care facilities and other crowded settings. In recent years several outbreaks of Norovirus gastroenteritis in schools have been reported in China [11, 12]. Guangzhou is a city in Guangdong Province, southern China, with millions of floating workers and residents. There were more than two outbreaks in four schools from 2015 to 2017 in Guangzhou according to the National Public Health Emergency Event Surveillance System (PHEESS). This suggests problems in the prevention and control of intestinal infectious diseases in local schools.

On March 8, 2018, The Center for Disease Control and Prevention of Guangzhou (GZCDC) was notified that a large number of students at a school in Guangzhou city developed sudden onset of diarrhea and vomiting; the event attracted media attention. To control the outbreak, we immediately formed a team to conduct an epidemiological investigation and to determine the causative agent, sources, role of transmission and risk factors of the infections.

## Methods

### Epidemiological investigation

In the investigation of this outbreak, the study population consisted of probable and confirmed cases. Probable cases were defined as teachers and students in the school with at least one of the following three symptoms: 1) diarrhea (≥ three times/day accompanied by changes in stool properties); 2) diarrhea (< three times/day accompanied by changes in stool properties) and vomiting; 3) vomiting more than twice within 24 h since March 8, 2018. Confirmed cases were those probable cases with positive test results for norovirus by reverse transcription polymerase chain reaction (RT-PCR). An active search was conducted for cases among all students, teachers and other staff members; cases were identified based on a uniform epidemiological questionnaire. Trained investigators participated in the survey. To analyze the potential transmission mode, a case control study was carried out using online standardized questionnaires. The control group was randomly selected healthy students of the same sex and age as the case, but without gastrointestinal symptoms. Data were obtained regarding demographic characteristics (sex, age, grade and class), symptoms (nausea, vomiting, diarrhea, abdominal pain and fever), personal hygiene habits, history of contact with a person who had diarrhea and/or vomiting and dining locations during the past 3 days. This study was approved by the Ethics Committee of Guangzhou center for disease control and prevention (GZCDC-ECHR-2017A0008).

### Specimen collection and laboratory tests

The following samples were collected for testing rectal swabs or stool samples from cases; rectal swabs from food handlers in the off-campus restaurant and canteen staff in the school; food scraps and environmental smear swabs (from cutting board, meat washing pond and so on) from the off-campus restaurant. Samples of drinking water were collected to detect intestinal viruses using RT-PCR, including Norovirus, Adenovirus and Rotavirus, as well as intestinal bacteria via culture, including *Salmonella, Shigella, enterohaemorrhagic Escherichia coli, Campylobacter, Staphylococcus aureus, Bacillus cercus, and Vibrio parahaemolyticus*. We used the Qiagen OneStep RT-PCR Kit to amplify the RNA-dependent RNA polymerase (RdRp) fragment of norovirus. The primer sequences were used in the previous studies [13, 14]. Five of the PCR products from cases, employees and environment samples were characterized by sequencing and phylogenetic analysis. After purification, the products were sent to Invitrogen (Shanghai, China) for sequencing. We download multiple genotype sequences from GenBank, and the cluster and neighbor-joining method was used to identify the genetic relationship between the norovirus and other strains from previous studies.

### Statistical analysis

Distributions of the major symptoms during the outbreaks were summarized using frequencies and proportions. Attack rates were calculated and compared using chi-square test between the two groups.

Combining the univariate analysis results together with professional knowledge, the risk factors were included in an unconditional logistic regression multivariate analysis model (the Wald test was introduced forward to screen variables). All statistical tests were two-sided, and *P* values < 0.05 were considered statistically significant. All of these analyses were performed using R 3.2.1 (The R Project for Statistical Computing, Vienna, Austria).

## Results

### Descriptive epidemiology

A total of 157 cases fit the definition of the probable case, including 20 cases of laboratory-confirmed norovirus infection between March 8 and March 22, 2018. Vomiting (61%) was the most common symptom, and 38% of cases had diarrhea, 27% of cases had abdominal pain, 19% of cases had fever (Table 1). All cases were mild and no hospitalization, severe illness, or deaths occurred. A total 379 samples were collected, including 27 rectal swab samples from cases, 169 and 151 rectal swab samples of food handlers in the off-campus restaurant and canteen staff in the school, respectively, and 32 environmental swabs (21 from canteen in school and 11 from off-campus restaurant).

**Table 1:** The distribution of major symptom of cases in the outbreak from March 8 through 22, 2018

Symptom	N	%
Vomiting	96	61
Diarrhea	60	38
Abdominal pain	42	27
Fever	30	19
Abdominal distention	11	7

#### Time distribution

**Fig. 1:**
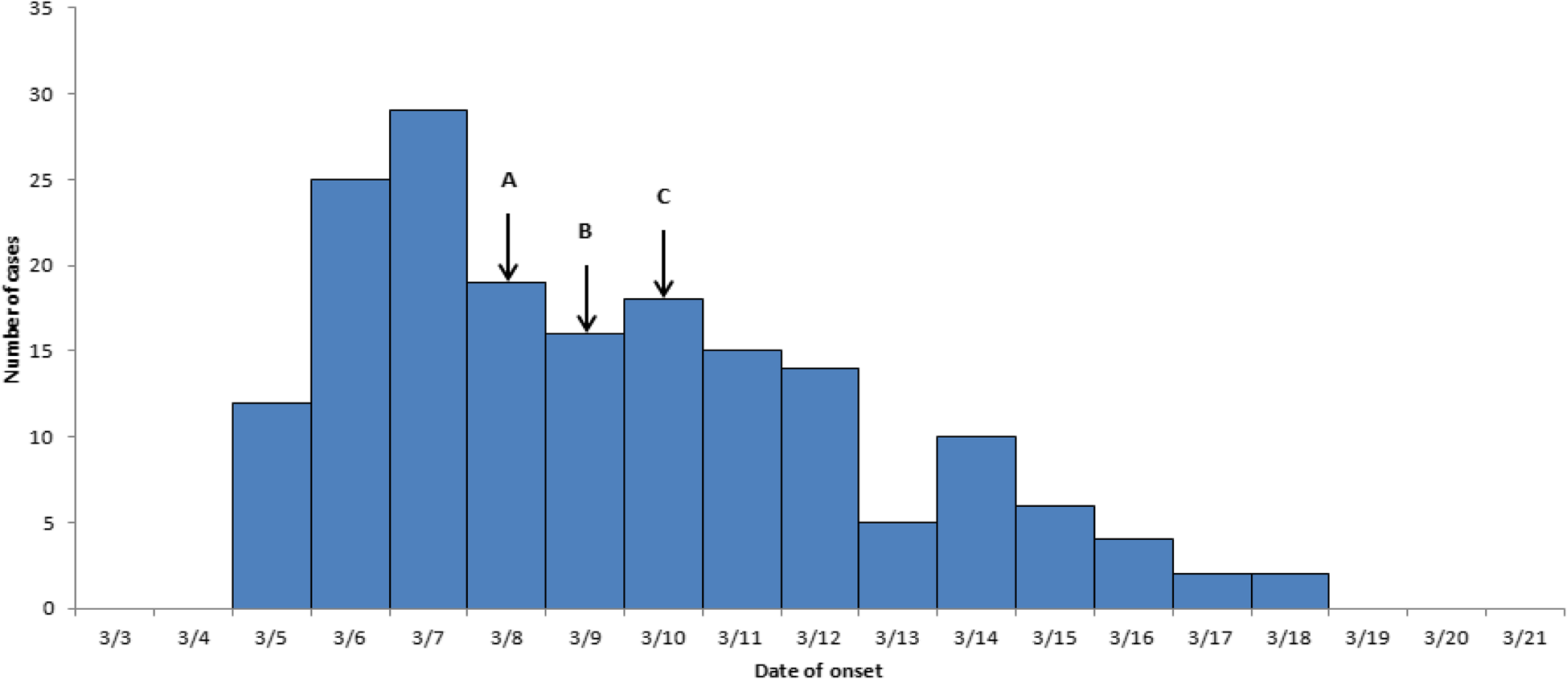
Time distribution of the onset of probable outbreak cases in Guangzhou, China. **a** Receiving the report and field epidemiology investigation. **b** Conducting the case-control study. **c** forbidden of off-campus eating and delivery service

The first case occurred on 5 March, four subsequent cases occurred on the same day. The peak incidence was reached on 6 March. After taking preventive and control measures, the number of cases decreased significantly. The epidemic curve with a sharp upward slope and a gradual downward slope described a point source outbreak (Fig. 1).

#### Space distribution

The distribution of cases in different departments and classes was scattered, with no clustering. There were cases in all 9 colleges, including in 85.7% (24/28) of student dormitory buildings and distributed among 114 dormitories. The four buildings with the largest number of cases were H (14 cases), B (11 cases), C (11 cases), A (8 cases), respectively. Seven dormitories had two cases or more.

#### Population distribution

All cases were students, aged 17–23 years old, including 47 males and 110 females. The incidence rates of male and female were 1.09% (47/4300) and 1.13% (110/9700), respectively. There was no significant difference between male and female (χ^2^ = 0.963, *P*> 0.05).

#### Case-control study

785 subjects were interviewed, including 157 probable cases and 628 control subjects. Univariate analysis and multivariable logistic regression analysis results are summarized in Table 2. The proportion of the students who had eaten delivery food three days before the onset of disease in the case group was 2.69 times that in the control group (95%*CI: 1.88–3.85*). Compared with control participants, risk factors included intake of take-out food 3 days earlier and, exposure to similar cases 72 h before onset and case in the same dormitory. Dining in the school canteen and washing hands with hand sanitizer or soap every time were protective factors.

**Table 2:** Univariate and multivariable logistic regression analysis of risk factors for acute gastroenteritis among students in Guangzhou, China

Variables	Cases (%)	Control (%)	single factor analysis		Multivariable logistic regression	
	(*N* = 157)	(*N* = 628)	OR	95%CI^#^	Adjusted OR	95%CI^#^
Had eaten delivery food 3 days ago	90(57.32)	209(33.28)	2.69**	1.88–3.85	1.96*	1.33–2.88
Dining place 1 day before						
Lunch						
Home	24(15.29)	65(10.35)				
School canteen	117(74.52)	546(86.94)	0.58*	0.35–0.97		
Delivery food	16(10.19)	17(2.71)	2.55*	1.11–5.83		
Midnight snack						
Home	29(18.47)	79(12.58)				
School canteen	107(68.15)	524(83.44)	0.65	0.38–1.10		
Delivery food	21(13.38)	25(3.98)	6.92**	3.49–13.73	6.96**	2.48–19.48
Dining place 2 days before						
Dinner						
Home	22(14.01)	74(11.78)				
School canteen	114(72.61)	523(83.28)	0.73	0.44–1.23		
Delivery food	21(13.38)	31(4.94)	2.28*	1.10–4.73		
Midnight snack						
Home	31(19.75)	93(14.81)				
School canteen	106(67.52)	505(80.41)	0.63*	0.40–1.00		
Delivery food	20(12.74)	30(4.78)	2	1.00–4.01		
Exposure history						
To similar cases 72 h before	26(16.56)	25(3.98)	4.78	2.68–8.55	2.94**	1.53–5.62
Have contact patient’s excrement	5(3.18)	4(0.64)	5.13*	1.36–19.23		
Short distance (≤1 m) contact or handling of excrement	10(6.37)	7(1.11)	6.02**	2.26–16.13		
Case in the same dormitory	46(29.30)	65(10.35)	3.59**	2.34–5.12	2.6**	1.61–4.21
Habit of washing hands						
No	1(0.64)	2(0.32)				
Occasionally	15(9.55)	36(5.73)	0.83	0.07–9.90		
Sometimes	43(27.39)	119(18.95)	0.72	0.06–8.17		
Every time	98(62.42)	471(75.00)	0.42	0.04–4.64		
With hand sanitizer or soap						
No	11(7.01)	18(2.87)				
Occasionally	81(51.59)	306(48.73)	0.43*	0.20–0.95		
Sometimes	32(20.38)	81(12.90)	0.65	0.28–1.52		
Every time	33(21.02)	223(35.51)	0.24	0.11–0.558	0.33*	0.13–0.83

*: *p* < 0.05;**: *p* < 0.01

#: 95%CI is 95% certificated interval

### Laboratory tests

A total of 20 rectal swab samples from students tested positive for Norovirus (GII) by RT-PCR, all the samples from food handlers and canteen environment were tested negative for Norovirus (Table 3).

**Table 3:** The frequency distribution of samples in the outbreak from March 8 through 22,2018

Samples	N	Positive rate (%)
Rectal swab samples		
Cases	27	74.07
Canteen employees	151	0.00
Out-campus restaurant employees	169	5.92
Environmental swab samples		
Canteen	21	0.00
Out-campus restaurant	11	18.18

**Fig. 2:**
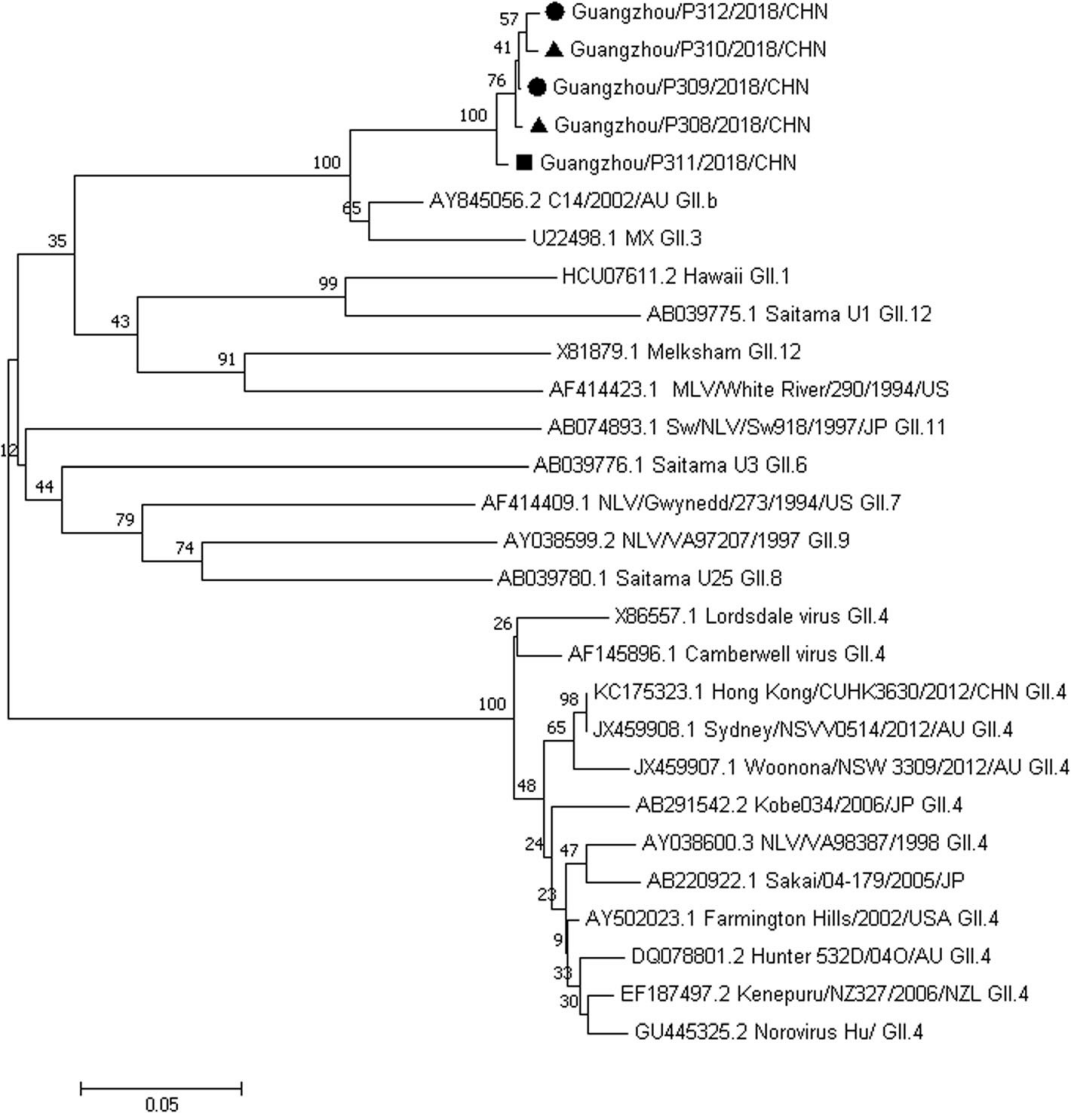
Phylogenetic tree of the nucleotide sequences of Noroviruses isolated from samples collected from patients, food handlers, and canteen environment. The phylogenetic tree was constructed using the neighbor-joining method, and the number at each branch point indicates the bootstrap value. Norovirus references selected from GenBank are indicated by accession numbers

After confirming Norovirus as the organism responsible for the outbreak, we collected rectal swab samples from employees and environmental swabs from the offcampus restaurant offering delivery service to students. Of 169 rectal swabs, 10 (5.92%) were positive for Norovirus, and 2 of 11 environmental swab samples were positive for Norovirus. All samples were negative for other viruses and bacteria. Phylogenetic analysis indicated that the Norovirus strains detected in five samples (two confirmed cases, a food handler, and two environmental swab samples) were all Norovirus GII.3 reference strains (Fig. 2).

### Environmental investigation

The College has four dining halls for teachers and students, distributed in four districts, with 151 employees. The operation mode is outsourcing bidding system. Four canteens provide breakfast, lunch, dinner and midnight snack. The catering and health service licenses and employees’ health certificates are complete. The tableware is mainly disinfected by heat and infrared ray. Chefs were monitored daily for health status, and there were no recent absentees who had complaints of gastrointestinal discomfort, such as diarrhea and vomiting.

The water used in the college is municipal tap water and direct drinking water. Recent water quality testing reports have all met the eligible standards.

## Discussion

According to the clinical symptoms, epidemiological characteristics and laboratory results, we clarified details of an outbreak of Norovirus infectious diarrhea. The epidemic curve with a sharp upward slope and a gradual downward slope described a point source outbreak. The entire school used the same municipal tap water and drinking water, and all laboratory tests were up to standard; therefore, water-source factors could be excluded. Having eaten takeout food was the main risk factor. More than 2 cases occurred in 7 dormitories. Exposure to similar cases 72 h before onset and the existence of case in the same dormitory were risk factors, and the time difference between the onset of the illness was as long as 10 days, which indicate that contact transmission from person to person could not be excluded in the later period of the epidemic. Moreover, we detected Norovirus GII.3 strain in environmental swab samples from off-campus restaurant, as well as in rectal swabs from food handlers using sequence analysis, thereby presenting a complete chain of evidence. The outbreak was quickly managed after the restriction of off-campus dinning and implementation of thorough disinfection and case isolation measures, which corroborated the judgment of foodborne and contact transmission in the outbreak.

Fecal-oral spread is the primary route of norovirus transmission, and humans can also be infected by direct person-to-person contact, consumption of contaminated food or water, or contact with contaminated environmental surfaces [15]. Foodborne transmission is an important source in the global spread of Norovirus [16, 17] and can occur through on-site contamination of food by food handlers or during the earlier steps of food production [15, 18]. In the period 2009–2012 [8], 48% (1008/2098) of the foodborne outbreaks were caused by Norovirus, among which infectious food workers were implicated as source of contamination in 364 (364/1008, 36%) cases in the United States.

Poor handling practices among infected food handlers are a common infection source in foodborne outbreak in Guangzhou. Foodborne transmission accounted for 40.74% of total Norovirus outbreaks in Guangzhou from 2016 to 2018, among which contamination by kitchen workers accounted for a large proportion. Personal hygiene practices among infected food handlers are considered the most important contributing factor in the transmission of foodborne diseases [19].

Two studies of food handlers have suggested that the Norovirus infectious rate of asymptomatic food handlers ranges from 1.0 to 3.7% [19, 20]. Foods may be contaminated by unhygienic manipulation by a food handler excreting the virus [20], which may probably be underestimated, because it is difficult to prove [21].

Given the importance of food handling in the prevention of Norovirus infections, food handlers should be advised to take special care to follow good kitchen hygiene practices, particularly hand washing. The epidemic season of Norovirus was winter in Guangzhou, southern China. Previous studies [19] suggested that the detection rate of asymptomatic infection in winter (2.20%) was higher than in nonwinter (0.16%). Good kitchen hygiene practices should be promoted, such as correct and frequent hand washing. Food handlers with gastrointestinal symptoms such as vomiting and diarrhea should be restricted to continue working, especially in the winter. Furthermore, daily health monitoring of food handlers and cleaning and disinfection of the environment are of great importance.

Periodic monitoring with viral pathogens such as Rotavirus and Norovirus has been performed to assess infection status in the general population [22]. However, epidemiologic surveillance data on food handlers in or out of schools are scarce in China. Enhancement of the surveillance of gastrointestinal symptoms of food practitioners and timely detection and control of disease outbreaks are critical measures to reduce the impact of such events. Recently, we have taken the first step in monitoring the Norovirus infections in asymptomatic food handlers in two colleges before the new semesters in Guangzhou.

An analysis of many outbreaks identified Noroviruses of the GII genogroup as the most common strains worldwide, among which strain GII.4 has become predominant. In this study, we showed that Norovirus GII.3 was the causative agent, and the original route of transmission was foodborne which caused infection in the primary cases. From 2012 to 2015, the strain GII.3 accounted for 12.3% of the 73 Norovirus outbreaks in Guangdong province, China [23]. Previous studies have indicated that the prevalence of infecting genotypes differs according to different human populations and routes of transmission [15, 24]. Genotype GII.4 is more often associated with transmission mediated by person-to-person contact than with other types of transmission, whereas non-GII.4 genotypes, such as GII.3, are more often associated with foodborne transmission [16], a trait that may be related to the proposed theory that GII.3 strains have greater stability in food than GII.4 strains. These results suggest that during suspected food outbreaks which presents with gastroenteritis, GII.3 Norovirus should not be ruled out.

To the best of our knowledge, this is the first study to have identified Norovirus as the causative agent in a foodborne outbreak caused by consuming take-out food in China. Food delivery services have recently become common in China in recent years, and the hygienic condition of delivery food is not easy to control and cannot be guaranteed. The contamination of the food can occur in any stage, from food preparation to distribution, and food handlers must play an important role. The current results highlight the risk of contamination of takeout food contamination by Norovirus.

## Conclusion

Food handling practices carry potential risk of acute gastroenteritis outbreaks due to inadequate surveillance and supervision. More attention should be paid to the monitoring and supervision of food handlers to reduce the incidence of Norovirus-related acute gastroenteritis associated with delivery food.

## Data Availability

The data that support the findings of this study are available from the National Public Health Emergency Event Surveillance System (PHEESS) but restrictions apply to the availability of these data, which were used under license for the current study, and so are not publicly available. Data are however available from the authors upon reasonable request and with permission of PHEESS.

## Abbreviations

CI: Confidence interval
G: Genogroup
RT-PCR: Reverse transcription polymerase chain reaction
